# Progress in Surgery of the Stomach

**Published:** 1901-08-03

**Authors:** 


### Progress in Surgery of the Stomach.

**Total Extirpation of the Stomach** has been recently performed by Bardeleben.[1] The patient, a woman aged 52 years, had suffered for a year previously from pain in the stomach, accompanied by nausea and eructation but without vomiting, and she lost 40 lb. in weight. A movable tumour of the size of a fist could be felt to the left of the umbilicus. On opening the abdomen the whole anterior wall of the stomach from the cardia to the pylorus was found to be densely infiltrated. The œsophagus was thereupon divided below a temporary ligature placed close to the diaphragm, and the stomach—inclusive of both cardia and pylorus—was removed with detachment of the great and small omenta. The orifice of the duodenum was sutured, and the end of the œsophagus was attached laterally to the small intestine some distance lower down. The operation lasted 75 minutes, and the subsequent course of the case was quite uneventful. All rectal alimentation was discontinued after the third day. Microscopical examination showed the tumour to be colloid cancer.

**Cancer of the Stomach.**—Operative treatment is advocated by Meriwether,[2] who lays great stress on the importance of early diagnosis. The presence of a tumour is not necessary for diagnosis, and Czerny, Kraske, and others insist that when a tumour is distinguishable a radical operation is out of the question. Hartmann[3] discusses the question of diagnosis and treatment of cancer of the stomach. The lymphatic distribution for the stomach follows three main systems: 1. The superior or coronary system drains the greater part of the anterior and posterior surfaces and all the lesser curvature, the collecting glands being situate along this curvature with the coronary vessels. 2. The inferior system follows the distribution of the right gastro-epiploic artery and ends in the glands above and behind the pylorus. The left or splenic system follows the splenic vessels and ends in glands in the gastro-splenic omentum. The author's opinion is much in favour of the posterior method of gastro-enterostomy by suture alone, and he has seen no accidents follow the division of the gastro-colic omentum. With reference to partial or complete gastrectomy he says that the surgeon must be sure of the mobility of the section involved before any resection is undertaken. For this assurance a finger-opening should be made through the gastro-hepatic omentum above, and the gastro-colic omentum below, the pylorus, so that the state of the prevertebral glands and the question of adhesions between the stomach and the pancreas can be definitely settled. After resection of the growth, the anastomosis should never be end to end. The stomach wound should be closed, and, if the duodenum be freely movable, this may be sutured to a new opening on the posterior and under surface of the stomach. If the duodenum be fixed, then both it and the stomach should be closed and a new gastro-enterostomy made. Mayo[4] says that ascites, even in a limited degree, contra-indicates any plastic operation such as gastro-enterostomy, for firm adhesions do not take place. Macdonald[5] considers that any one or a combination of the following symptoms is a sufficient indication for operation:—(1) A chronic gastritis which is progressive in character under proper dietetic, medicinal, and physical treatment; (2) a loss of gastric motility; (3) progressive diminution of gastric peristalsis; (4) a diminution of free hydrochloric acid, progressive in character; (5) emaciation of the patient under forced diet; (6) reduction of the hæmoglobin in the blood, progressive to 65 per cent. or under, and a moderate leucocytosis. He quotes Guinard's statement that in 148 cases of pylorectomy with end-to-end anastomosis there were 37·8 per cent. of deaths, and in 64 cases of pylorectomy with subsequent lateral anastomosis there had been 15 6 per cent. of deaths. King[6] records a successful case of pylorectomy on a man in his seventy-first year.

**Hour-glass Stomach.**—Moynihan[7] says that the evidence of the existence of hour-glass stomach as a congenital deformity is insufficient to be convincing. Acquired hour-glass stomach may be caused by: (1) Perigastric adhesions; (2) ulcer, with local perforation and anchoring to the anterior abdominal wall; (3) circular ulcer with subsequent cicatricial contraction and induration; (4) cancer. The symptoms of hour-glass stomach are most often those of dilated stomach supervening upon chronic ulcer of the stomach. In most cases the dilated stomach has led to operation for the relief of supposed pyloric stenosis. In certain cases, however, the symptoms and signs are clear and characteristic. Two of the most helpful signs were pointed out by Wölfler: 1. A phenomenon observed upon washing out the stomach. The fluid introduced into the stomach seems to disappear altogether, "as though it had flowed through a large hole," and is not returned through the tube. In such a circumstance the fluid passes, it is obvious, from the one compartment to the other. 2. It was further noticed that when the stomach was washed out until the lotion returned clear a sudden unlooked-for gush of foul or even putrid fluid occurred; or if, after gentle lavage until the stomach seemed clean, an interval of a few minutes was allowed to elapse, and the tube again passed, foul or dirty fluid at once returned. This is due, doubtless, to the reflux of the contents of the pyloric cavity through the stricture. In some cases the bubbling and gushing of fluid through a narrow chink has been heard with the stethoscope on distending the stomach with carbonic acid gas. The various operative procedures which may be adopted for this condition are as follows:—(1) Gastroplasty—an adaptation to the body of the stomach of Heineke's operation for pyloric stenosis. (2) Gastro-gastrostomy or gastro-anastomosis. (3) Gastro-enterostomy—an operation which almost necessarily leaves one of the "halves" of the stomach undrained. (4) Partial gastrectomy, which should always be the operation of choice in cases of cancer.

[1] Lancet, April 20, 1901, p. 1154, and Deut. Med. Woch., April 11. [2] Charlotte Med. Jour., Jan. 1901, p. 12. [3] Med. Chron., Feb. 1901, p. 363. [4] Ibidem, p. 366. [5] Med. Rec, Dec 1, 1900, p. 872. [6] Med. Rec., May 11, 1901, p. 730. [7] Lancet, April 27, 1901, p. 1190.

